# Ligand manipulation of charge transfer excited state relaxation and spin crossover in [Fe(2,2′-bipyridine)_2_(CN)_2_]

**DOI:** 10.1063/1.4985017

**Published:** 2017-06-06

**Authors:** Kasper S. Kjær, Wenkai Zhang, Roberto Alonso-Mori, Uwe Bergmann, Matthieu Chollet, Ryan G. Hadt, Robert W. Hartsock, Tobias Harlang, Thomas Kroll, Katharina Kubiček, Henrik T. Lemke, Huiyang W. Liang, Yizhu Liu, Martin M. Nielsen, Joseph S. Robinson, Edward I. Solomon, Dimosthenis Sokaras, Tim B. van Driel, Tsu-Chien Weng, Diling Zhu, Petter Persson, Kenneth Wärnmark, Villy Sundström, Kelly J. Gaffney

**Affiliations:** 1 PULSE Institute, SLAC National Accelerator Laboratory, Stanford University, Menlo Park, California 94025, USA; 2 Centre for Molecular Movies, Department of Physics, Technical University of Denmark, DK-2800 Lyngby, Denmark; 3 Division of Chemical Physics, Department of Chemistry, Lund University, Box 124, SE-22100 Lund, Sweden; 4 Department of Physics, Beijing Normal University, Beijing 100875, China; 5 LCLS, SLAC National Accelerator Laboratory, Menlo Park, California 94025, USA; 6 Department of Chemistry, Stanford University, Stanford, California 94305, USA; 7 SSRL, SLAC National Accelerator Laboratory, Menlo Park, California 94025, USA; 8 Deutsches Elektronen-Synchrotron (DESY), Photon Science, Notkestraße 85, 22607 Hamburg, Germany; 9 Max Planck Institute for Biophysical Chemistry, Am Faßberg 11, 37077 Göttingen, Germany; 10 HPSTAR, 1690 Cailun Rd., Pudong, Shanghai 201203, China; 11 Division of Theoretical Chemistry, Department of Chemistry, Lund University, Box 124, SE-22100 Lund, Sweden; 12 Center for Analysis and Synthesis, Department of Chemistry, Lund University, Box 124, SE-22100 Lund, Sweden

## Abstract

We have used femtosecond resolution UV-visible and Kβ x-ray emission spectroscopy to characterize the electronic excited state dynamics of [Fe(bpy)_2_(CN)_2_], where bpy=2,2′-bipyridine, initiated by metal-to-ligand charge transfer (MLCT) excitation. The excited-state absorption in the transient UV-visible spectra, associated with the 2,2′-bipyridine radical anion, provides a robust marker for the MLCT excited state, while the transient Kβ x-ray emission spectra provide a clear measure of intermediate and high spin metal-centered excited states. From these measurements, we conclude that the MLCT state of [Fe(bpy)_2_(CN)_2_] undergoes ultrafast spin crossover to a metal-centered quintet excited state through a short lived metal-centered triplet transient species. These measurements of [Fe(bpy)_2_(CN)_2_] complement prior measurement performed on [Fe(bpy)_3_]^2+^ and [Fe(bpy)(CN)_4_]^2−^ in dimethylsulfoxide solution and help complete the chemical series [Fe(bpy)_N_(CN)_6–2N_]^2N-4^, where N = 1–3. The measurements confirm that simple ligand modifications can significantly change the relaxation pathways and excited state lifetimes and support the further investigation of light harvesting and photocatalytic applications of 3*d* transition metal complexes.

## INTRODUCTION

Harnessing the optical and photocatalytic properties of transition metal complexes requires long-lived, metastable electronic excited states. Numerous 4*d* and 5*d* transition metal complexes exhibit long-lived charge transfer excited states,^1–6^ but the majority of complexes utilizing abundant 3*d* transition metals have very short excited state lifetimes or absorption predominantly in the UV.^7–16^ Unlike most 4*d* and 5*d* complexes, many 3*d* transition metal complexes have exchange and correlation energies of similar magnitude to the ligand field splitting energy. For these complexes, a number of ligand field excited states prove to be energetically accessible from the electronic excited states generated by optical excitation and strongly influence the non-radiative relaxation in 3*d* complexes.

The challenge of extending the electronic excited state lifetimes of 3*d* transition metal complexes can be recast as the challenge of controlling the energetics and dynamics of internal conversion and intersystem crossing.^17,18^ A series of ultrafast experimental studies have demonstrated that the traditional ordering of dynamical events in electronic excited states—intramolecular vibrational redistribution, followed by internal conversion, followed by intersystem crossing—does not accurately describe the relaxation dynamics of 3*d* transition metal complexes. An alternative framework is emerging, where the coupled, non-adiabatic dynamics of electrons and nuclei control the rate of electronic excited state relaxation.^19^ Within this framework, two goals emerge: (1) identify the location of conical intersections and seams between electronic states and the excited state trajectories that sample these regions of phase space and (2) determine how to inhibit the accessibility of these intersections and seams from the Franck-Condon region of optically allowed electronic excited states.^20–25^

These two goals have motivated our studies of the ultrafast electronic state relaxation dynamics in [Fe(bpy)_N_(CN)_6–2N_],^2N-4^ where N = 1–3.^26,27^ In this series of molecules, the metal-to-ligand charge transfer (MLCT) state is the lowest energy electronic excited state that can be accessed by an optically allowed transition. In [Fe(bpy)_3_]^2+^, optically induced spin crossover occurs within 200 fs. This photo-excited spin crossover involves two active electrons that undergo both internal conversion and intersystem crossing.^8–16^ The photo-induced spin crossover mechanism has been the focus of more recent measurements and theoretical calculations. Many,^12,15,28–30^ though not all,^10^ of these studies provide support for a stepwise spin crossover mechanism, where the MLCT excited state transitions to a metal-centered quintet (^5^MC) excited state through a metal-centered triplet state (^3^MC). The work of Chergui and Auboeck represents the most prominent case for direct MLCT relaxation to the ^5^MC state in [Fe(bpy)_3_]^2+^,^10^ though the ≤50 fs lifetime extracted from these UV-visible pump-probe measurements provides a poor fit to both ultrafast x-ray absorption near edge structure (XANES) and x-ray emission spectroscopy (XES) measurements.^12,15^

The large variations in the ligand field strength and symmetry of the [Fe(bpy)_N_(CN)_6–2N_]^2N-4^ series provide a coarse grained approach to changing MLCT excited state relaxation dynamics and pathway.^26,27,31–33^ We recently demonstrated that substituting two bpy ligands in [Fe(bpy)_3_]^2+^ with the four CN^−^ ligands to make [Fe(bpy)(CN)_4_]^2−^ leads to an MLCT excited state lifetime of 19 ps in aprotic solvents.^34^ The present study of [Fe(bpy)_2_(CN)_2_] complements our prior studies of [Fe(bpy)_3_]^2+^ and [Fe(bpy)(CN)_4_]^2−^ and represents an extension of our investigations of the MLCT relaxation dynamics in the [Fe(bpy)_N_(CN)_6–2N_]^2N-4^, where N = 1–3, a series of complexes. The motivation for these studies is the systematic identification of how symmetry, ligand field strength, and covalency dictate the dynamics and mechanisms of internal conversion and intersystem crossing in 3*d* transition metal complexes. The overall charge of the molecule changes, during the series, as well, which will influence the solvation dynamics. For [Fe(bpy)_2_(CN)_2_], the absence of a charge will influence the solvation dynamics of the molecule. The strong solute-solvent interaction between the cyano ligands^35^ and hydrogen bonding solvents has an even more significant effect on solvation in this series of complexes.^36^ The potential influence of solvation on the energetics and dynamics of internal conversion and intersystem crossing in the [Fe(bpy)_N_(CN)_6–2N_]^2N-4^ series of complexes warrants systematic investigation in the future.

Characterizing the dynamics and mechanisms of internal conversion and intersystem crossing requires robust identification of both the excited electronic states involved in the MLCT relaxation dynamics, as well as the vibrational trajectories that lead to the intersections and seams between electronic excited states that control the rate of non-adiabatic transitions between electronic states. Spectroscopically, we need to differentiate between the charge-transfer and ligand field electronic excited states that participate in the spin crossover and determine the rate with which they interconvert. We achieve this objective by combining two complementary probes of electronic relaxation dynamics: femtosecond resolution iron 3*p*–1*s* (Kβ) XES to measure the time evolution of the Fe spin moment^37–42^ and femtosecond UV-visible spectroscopy to track the decay dynamics for the MLCT excited state via the bpy anion excited state absorption. With this combination of x-ray and optical probes, we have determined that the MLCT excited state of [Fe(bpy)_2_(CN)_2_] undergoes spin crossover on the 200 fs timescale via a sequential mechanism, MLCT → ^3^MC → ^5^MC, analogous to spin crossover in photoexcited [Fe(bpy)_3_]^2+^.

## RESULTS AND DISCUSSION

**FIG. 1.**
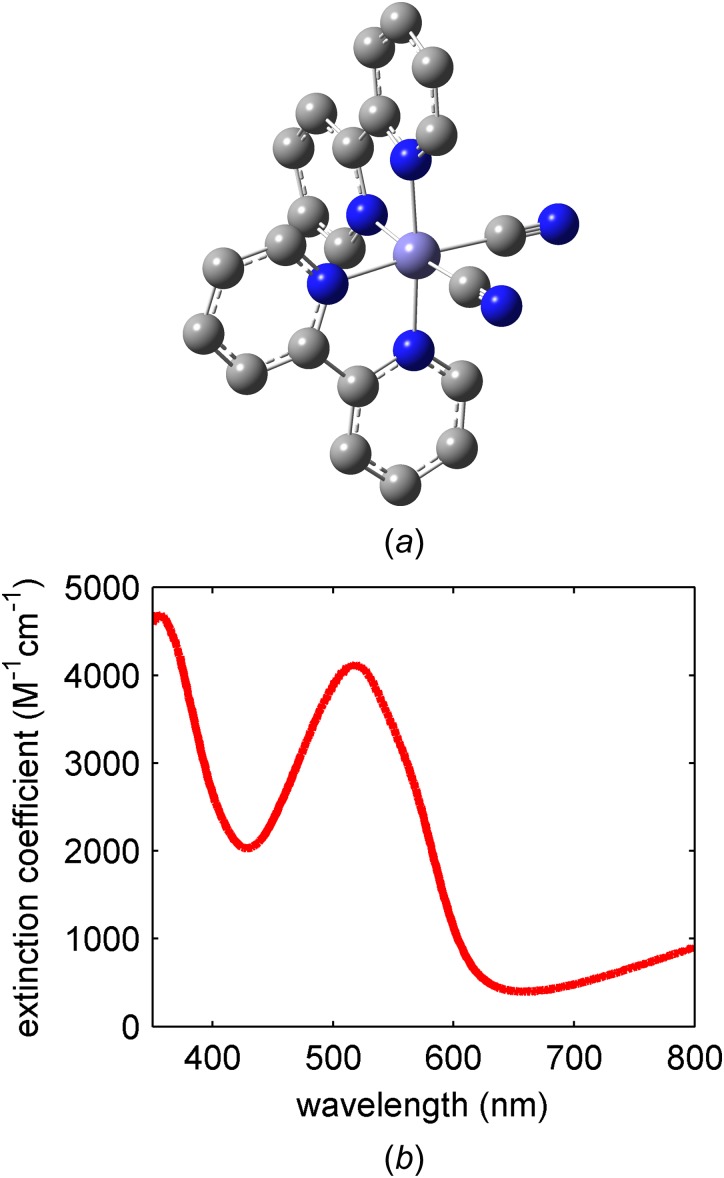
Molecular structure of investigated iron coordination complex (a) [Fe(bpy)_2_(CN)_2_]. Hydrogen atoms are not shown. (b) The UV-visible absorption spectrum of [Fe(bpy)_2_(CN)_2_] in methanol.

**FIG. 2.**
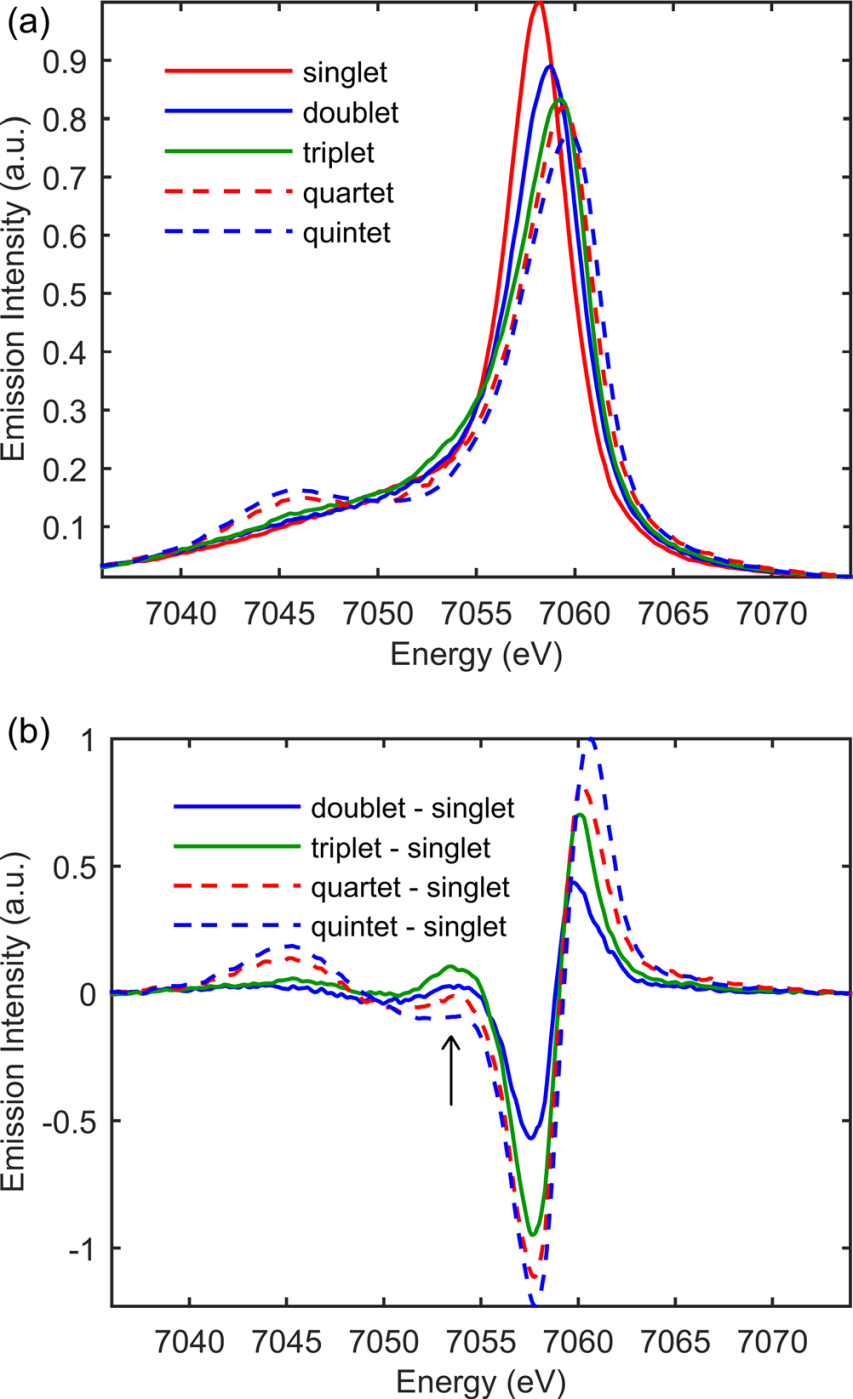
(a) The Kβ emission spectra of ground-state iron complexes with different spin moments: singlet ([Fe(bpy)_3_]^2+^, red), doublet ([Fe(bpy)_3_]^3+^, blue), triplet (iron(II) phthalocyanine, green), quartet (iron(III) phthalocyanine chloride, red dashed), and quintet ([Fe(phenanthroline)_2_(NCS)_2_], blue dashed). (b) Model complex difference spectra for the MLCT, ^3^MC and ^5^MC excited states constructed by subtracting the singlet model complex spectrum from the doublet, triplet and quintet model complex spectra shown in (a). Arrow horizontal position set to 7054 eV. Adapted with permission from Zhang *et al.*, Chem. Sci. **8**, 515 (2017). Copyright 2017 Royal Society of Chemistry.

Figure 1(b) shows the UV-visible absorption spectrum of [Fe(bpy)_2_(CN)_2_], which we prepared using the published procedure.^43^ Fe Kβ x-ray emission arises from 3*p* filling of the 1*s* hole. The strong exchange interaction between electrons in the 3*d* and 3*p* levels makes Kβ x-ray emission spectroscopy (XES) sensitive to the 3*d* spin moment.^37–42^ This sensitivity can be seen in Fig. 2(a), where the Fe Kβ emission spectra for a variety of Fe compounds with distinct spin moments have been plotted. By subtracting the singlet reference spectrum from the remaining reference spectra, the characteristic difference spectra generated by a change in Fe spin-moment can be constructed and are shown in Fig. 2(b). The dominant source of spectral variation results from variation in spin state, making Kβ XES an excellent probe of spin dynamics prior to the onset of single shot x-ray damage observed at x-ray laser sources.^44^ We use the electronic ground state spectra shown in Fig. 2(a) as the model spectra for the possible excited state charge and spin state configurations of [Fe(bpy)_2_(CN)_2_]. As can be seen in Fig. 2(b), the magnitude and shape of the difference spectra constructed from these references provide key signatures for the MLCT excited state, ^3^MC excited states, and ^5^MC excited states. As discussed by Zhang *et al.* in the context of spin crossover in [Fe(bpy)_3_]^2+^,^12^ the difference signal centered at 7054 eV has particular importance, because ^3^MC excited states give an increased emission at this energy, while ^5^MC excited states show a decrease in emission signal. Using ground state spectra to model the excited state spectra of distinct molecules does have limitations that must be considered when choosing the model complexes. A variety of measurements and calculated spectra have demonstrated that the Kβ spectrum shows little sensitivity to molecular symmetry for equal spin states,^39,40,45^ but the covalency of the metal-ligand bond does have an impact on the spectrum.^42^ This is demonstrated most clearly for high spin ferric iron complexes at the extremes of metal-ligand covalency^42^ because the Kβ spectrum only reflects the Fe contribution to the spin moment. This aspect of the Kβ XES adds to the information content of the technique but also means that molecules with similar coordination bonding need to be chosen to model excited state spin dynamics. The experimental details can be found in the supplementary material.

**FIG. 3.**
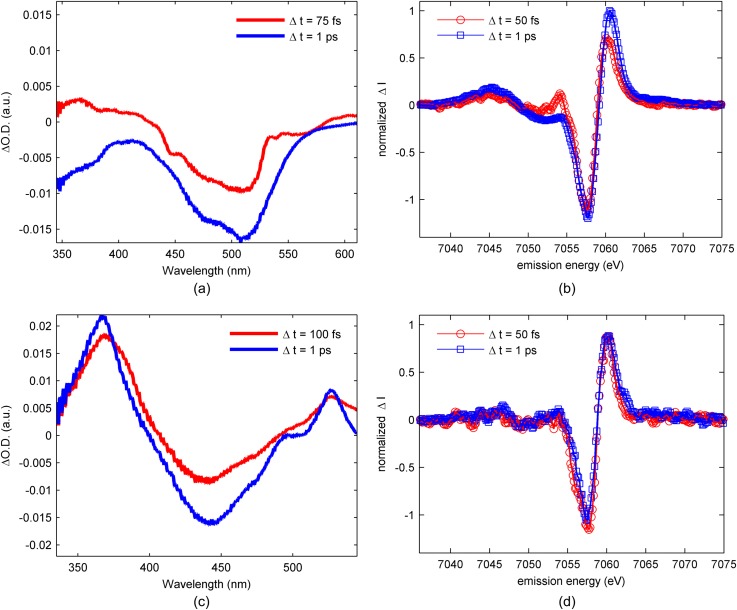
(a) Transient UV-visible absorption spectra obtained at 75 fs time delay (red curve) and 1 ps time delay (blue curve) for [Fe(bpy)_3_]^2+^ in water. (b) Kβ transient difference spectra obtained at 50-fs time delay (red circles) and 1-ps time delay (blue square) for [Fe(bpy)_3_]^2+^ in water. (c) UV-visible pump-probe difference spectrum at 75 fs (red curve) and 1 ps (blue curve) for [Fe(bpy)(CN)_4_]^2-^ in dimethyl sulfoxide. (d) Kβ transient difference spectra obtained at 50 fs time delay (red circles) and 1 ps time delay (blue square) for [Fe(bpy)(CN)_4_]^2-^ in dimethyl sulfoxide. (a) and (b) Adapted with permission from Zhang *et al.*, Nature **509**, 345 (2014). Copyright 2014 Nature Publishing Group. (c) and (d) Adapted with permission from Zhang *et al.*, Chem. Sci. **8**, 515 (2017). Copyright 2017 Royal Society of Chemistry.

We use the complementary attributes of UV-visible absorption and Fe Kβ x-ray emission spectroscopy to track the charge and spin dynamics induced by photo-excitation. In our pump-probe measurements, we have photo-excited the molecule in the lowest energy MLCT excited state. Figure 3(a) shows the UV-visible pump-probe signal of [Fe(bpy)_3_]^2+^ and Fig. 3(c) for [Fe(bpy)(CN)_4_]^2−^ at the time delays of 75 fs and 1 ps. For the 1 ps time delay spectra, the strong excited state absorption at 370 nm appears for [Fe(bpy)(CN)_4_]^2−^ and not [Fe(bpy)_3_]^2+^. This absorption is associated with the 2,2′-bipyridene radical anion and provides a clear signature of the MLCT excited state.^46,47^ Figure 3(b) shows the Fe Kβ XES pump-probe difference signal for [Fe(bpy)_3_]^2+^^12^ and Fig. 3(d) for [Fe(bpy)(CN)4]^2−^ at pump-probe time delays of 50 fs and 1 ps.^34^ The sensitivity of the Kβ emission spectrum to the Fe spin moment allows us to monitor the presence of metal centered excited states and provides an additional monitor for the MLCT excited state since electron transfer changes the Fe spin moment from *S* = 0 to *S* = 1/2. The combination of these methods enables a clear interpretation of the MLCT relaxation mechanism in [Fe(bpy)_2_(CN)_2_].

**FIG. 4.**
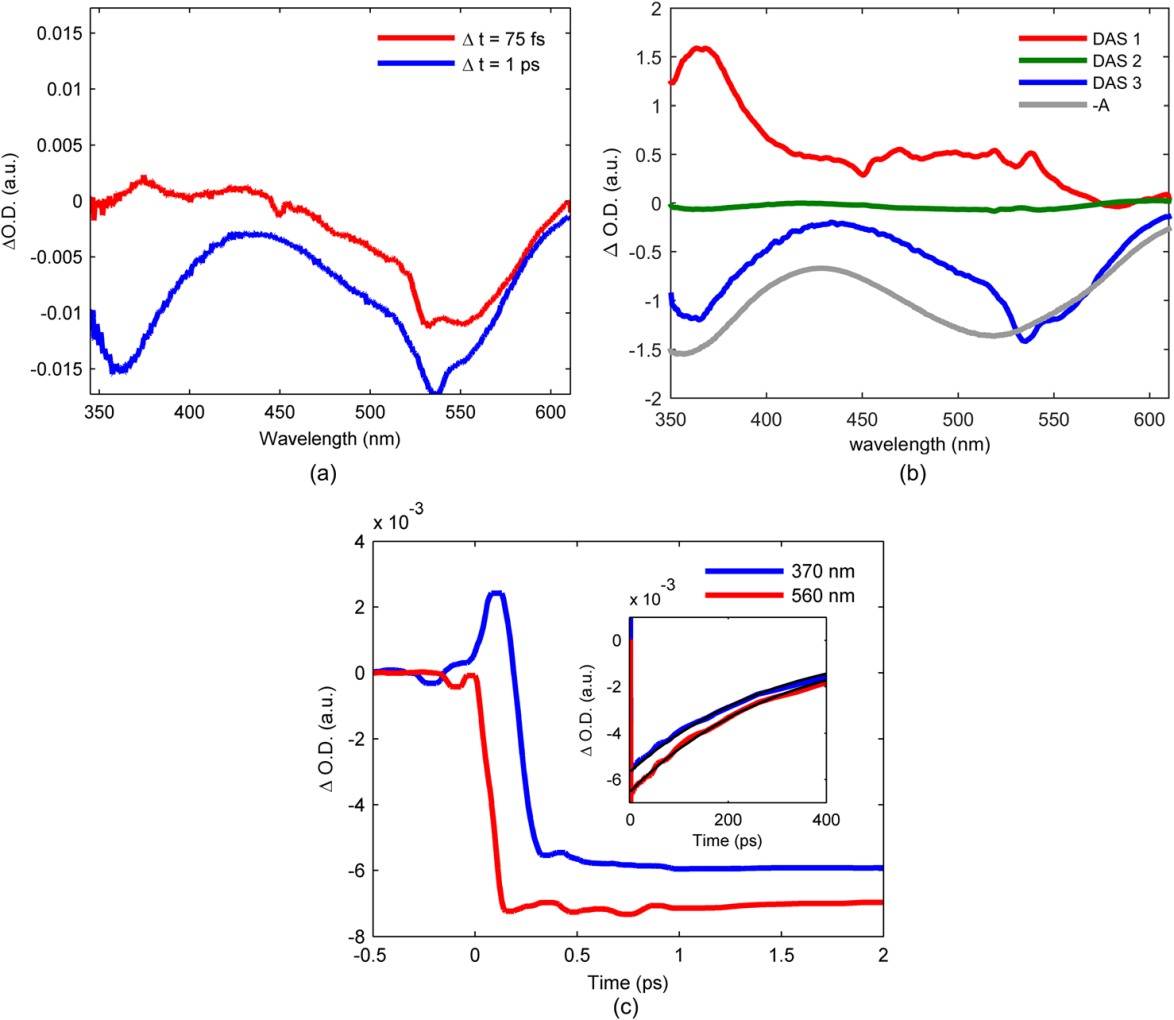
(a) Transient UV-visible absorption spectra obtained at 75-fs time delay (red curve) and 1-ps time delay (blue curve) for [Fe(bpy)_2_(CN)_2_] in methanol. (b) The three decay associated spectra returned by global analysis of the data presented in panel (a) (red, green and blue curves) and inverted ground state UV visible absorption spectrum (gray curve). (c) Kinetics of the UV visible absorption data at 370 and 560 nm (red and blue curves). Inset shows the signal at long time scales with single-exponential fits (black curves) retuning a 256 ± 4 ps lifetime.

**FIG. 5.**
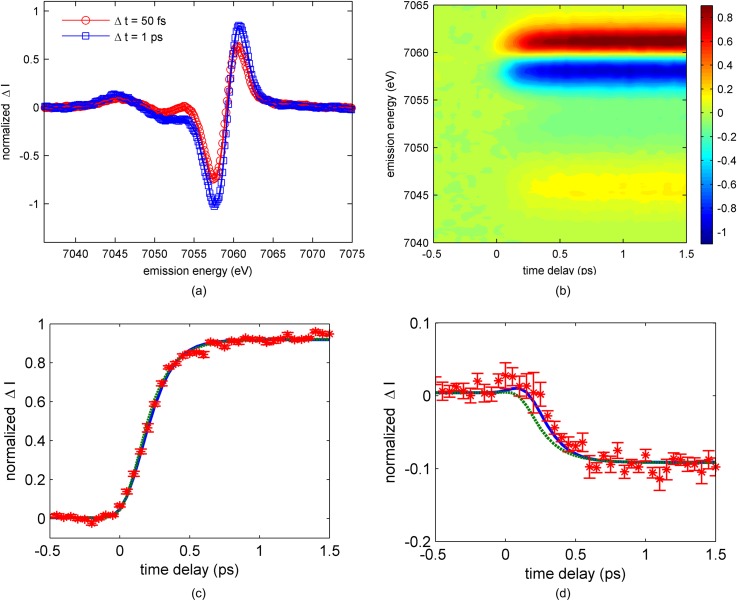
(a) Kβ transient difference spectra obtained at 50 fs time delay (red circles) and 1 ps time delay (blue square) for [Fe(bpy)_2_(CN)_2_] in methanol. (b) Time-dependent optically induced two-dimensional Kβ fluorescence difference spectra for [Fe(bpy)_2_(CN)_2_] in methanol. (c) and (d) The difference signal measured at a Kβ emission energy of 7,061 eV (b) and 7,054 eV (c) for [Fe(bpy)_2_(CN)_2_] in methanol (red stars), as well as the best fit achieved for kinetic models with (blue) or without (green dashed) a ^3^MC transient. The error bars in (b) and (c) reflect the standard error for the difference signal determined from six independent measurements.

Figure 4(a) shows the UV-visible difference spectra measured at the time delays of 75 fs and 1.0 ps, and Fig. 5(a) shows the Kβ difference spectra measured at 50 fs and 1.0 ps time delays for 25 mM [Fe(bpy)_2_(CN)_2_] dissolved in methanol and photo-excited at 550 nm. Consistent with [Fe(bpy)_3_]^2+^,^12^ the sub-100 fs spectra show the expected signatures for the MLCT excited state in both the UV-visible and Kβ difference spectra. The 1.0 ps time delay Kβ spectrum in Fig. 5(a) provides clear evidence of ultrafast spin crossover in [Fe(bpy)_2_(CN)_2_]. The dynamics and mechanism of the spin crossover reaction will be discussed below.

We use different approaches to analyze the UV-visible and Kβ XES results. These include a principle component analysis framework based on singular value decomposition of the UV-visible difference spectra^48^ and model complex difference spectra for the Kβ emission difference spectra. Details of the data analysis can be found in the supplementary material. Global analysis of the principle components returns decay associated spectra (DAS). The DAS for [Fe(bpy)_2_(CN)_2_] can be found in Fig. 4(b). When the DAS can be assigned to specific molecular species or excited states, the time dependent amplitudes of the DAS provide a powerful means of characterizing excited state kinetics; distinguishing between spectral dynamics associated with changes in population from those associated with intramolecular vibrational redistribution and solvation can prove challenging. This weakness can be mitigated by thoughtful inspection of the component difference spectra and comparison to complementary transient measurements. We use a kinetic model based method for analyzing the Kβ emission difference spectra. This employs model spectra to analyze the Kβ difference spectra, rather than principle component analysis, because the amplitude of the difference spectra, not just the spectral profile, is critical in distinguishing between different spin states.^12^ This approach can potentially introduce bias in the analysis through the choice of model spectra and the kinetic model. We address this potential weakness by constructing distinct kinetic schemes and using statistical analysis to identify the scheme most consistent with the experimental difference spectra.

We consider two distinct mechanisms for the return of the photo-excited MLCT state to the electronic ground state: (1) relaxation of the MLCT excited state to the ground state via a ^5^MC excited state, where the quintet state is formed directly from the MLCT, and (2) relaxation of the MLCT excited state to the ground state via a ^5^MC excited state, where the MLCT decays to a transient ^3^MC before forming the ^5^MC state

MLCT→k1M5C→k3G, (1)

MLCT→k1M3C→k2M5C→k3G. (2)

The representative differential equations and their solution can be found in the supplementary material.

Figure 4 presents a subset of the UV-visible pump-probe data and analysis for [Fe(bpy)_2_(CN)_2_]. The full data set and analysis can be found in the supplementary material. DAS1 in Fig. 4(b) captures the excited state absorption feature at 370 nm found in the 75 fs time delay spectrum in Fig. 4(a). The increase in absorption at 370 nm has been associated with the 2,2′-bipyridine radical anion absorption, making this absorption feature a signature for the MLCT excited state, and the 120 ± 30 fs decay constant for DAS1 provides a measure of the MLCT excited state lifetime. Figure 4(c) shows the time dependent amplitude of the pump-probe signal at 370 nm, where the ultrafast decay of the positive signal around time zero reflects the short lifetime of the MLCT excited state. Following the decay of DAS1, DAS3 dominates the UV-visible pump-probe signal. The relatively weak amplitude of DAS2 suggests that it describes vibrational cooling of the system, and the associated lifetime of 1.5 ps, matches well with the 2.5 ps vibrational cooling component identified in [Fe(bpy)(CN)_4_]^2–^.^34^ DAS3 strongly resembles the ground state absorption spectrum and is therefore assigned to ground state bleach. This clearly demonstrates the MLCT excited state decay populates a persistent intermediate, but the absence of any excited state absorption features in the spectrum does not allow the nature of the long-lived intermediate to be determined from the UV-visible difference spectra. The Kβ emission difference spectra, however, allow us to definitively assign the persistent excited state signal to an intermediate ^5^MC excited state.

We use the same analysis approach for the Kβ emission difference spectra developed for the [Fe(bpy)_3_]^2+^ to analyze the [Fe(bpy)_2_(CN)_2_] data.^12^ The time-resolved difference spectra can be found in Figures 5(a) and 5(b), while the model fit of the difference spectra can be found in the supplementary material. Table I lists the parameters extracted from the best fit of the experiment to schemes (1) and (2). Given the obvious presence of photo-induced spin crossover in Fig. 5(a), we only fit the difference spectra to the two models involving ^5^MC formation before returning to the electronic ground state: the one where the MLCT decays directly to a metal centered quintet state represented by scheme (1) and the other where the MLCT relaxes to a ^5^MC via a ^3^MC transient represented by scheme (2). Figures 5(c) and 5(d) show the time-dependent difference signal measured at two x-ray emission energies: 7061 eV, where the difference signal is largest in Fig. 2(b), and 7054 eV, where the triplet model complex has a spectral signature clearly distinct from the MLCT and ^5^MC states, as shown in Fig. 2. The fits in Figs. 5(c) and 5(d) have been determined from a global analysis of the full time-dependent spectra, which can be found in the supplementary material. The statistical significance of the more complex kinetic model involving the triplet transient can be determined from an F-test comparison of the two models, as described in the supplementary material. The reduction in residuals achieved with the model containing the triplet transient is sufficient to reject the direct MLCT→^5^MC model with greater than 95% confidence.

**TABLE I.** Time-dependent Kb emission spectra of [Fe(bpy)_2_(CN)_2_] in methanol fit with two different kinetic models.

Kinetic model	Lifetime	Lifetime	Time zero	Instrument response (fs)	
	1/k_1_ (fs)	1/k_2_ (fs)	t_0_ (fs)	σ (fs)	Fwhm (fs)
With ^3^MC intermediate	120 ± 30	60 ± 20	0 ± 20	80 ± 10	180 ± 20
Without ^3^MC intermediate	150 ± 40		10 ± 15	76 ± 10	180 ± 20

The successful analysis of the experimental data relies on two constraints presented by the model spectra shown in Fig. 2 and two constraints derived from the kinetic models. We calibrate the spectrum and relative amplitudes of the difference signals for the MLCT, ^3^MC, and ^5^MC electronic excited states to match those of the model complex difference spectra. We also require all x-ray emission energies to be fit with a single time zero and all MLCT excited states to undergo spin crossover. Inspection of the time resolved Kβ difference spectra rules out the formation of any long lived concentration of triplet states and confirms [Fe(bpy)_2_(CN)_2_] undergoes complete spin crossover to a ^5^MC excited state on a time scale similar to [Fe(bpy)_3_]^2+^. Determining whether spin crossover occurs directly or through a ^3^MC transient requires the full data set to be fit in the same manner as that used for [Fe(bpy)_3_]^2+^ (Ref. 12) and is described in the supplementary material. For the fit to the direct spin crossover mechanism shown in Fig. 5(c), the fast rise in signal at 7061 eV requires a fast rise in ^5^MC population. As shown in Fig. 5(d), the fast rise in the direct mechanism fit at 7061 eV also leads to a fast drop in signal at 7054 eV because the ^5^MC state has a negative difference signal at 7054 eV. For the fit to the sequential spin crossover mechanism also shown in Fig. 5(c), the fast rise in signal at 7061 eV can be accommodated initially by a rise in ^3^MC population. Since the ^3^MC state does not have a negative difference signal at 7054 eV, the fast rise in ^3^MC population does not lead to a fast drop at 7054 eV. The stepwise transition through the ^3^MC excited state leads to a delayed onset of the drop in emission amplitude at 7054 eV relative to the rise in signal at 7061 eV, consistent with the experimental data. For the direct model, a shift in time zero to fit the data in Fig. 5(d) would lead to a poor fit of the data in Fig. 5(c).

The legitimacy of the kinetic model used in this analysis and previously for [Fe(bpy)_3_]^2+^ has been brought into question in the recent ultrafast K-edge x-ray absorption near edge spectroscopy (XANES).^15^ While this study supports the exponential decay of the MLCT excited state, the time-dependent expansion of the symmetric Fe-N bond length observed following the decay of the MLCT state cannot be reconciled with a ^3^MC state that decays exponentially. The XANES measurement can be best explained with a model involving ballistic transport through a ^3^MC transient where the transition from the ^3^MC to the ^5^MC state occurs over a narrow range of Fe-N symmetric stretch bond lengths.^15^ Unpublished simultaneous ultrafast XES and x-ray diffuse scattering (XDS) measurements with improved time resolution strongly support this conclusion. These new measurements for [Fe(bpy)_3_]^2+^ support the general mechanism proposed by Zhang *et al.*,^12^ but a dynamical, rather than kinetic, model for the relaxation process is needed for [Fe(bpy)_3_]^2+^. A similar conclusion seems plausible for [Fe(bpy)_2_(CN)_2_]. Simultaneous ultrafast XES and XDS measurements and ultrafast XANES measurements would clarify these issues.

## CONCLUDING REMARKS

A combination of femtosecond resolution UV-visible and Kβ emission spectroscopy has allowed the robust characterization of the electronic excited state dynamics of [Fe(bpy)_2_(CN)_2_]. Based on the experimental data and analysis, we conclude [Fe(bpy)_2_(CN)_2_] undergoes ultrafast spin crossover to a ^5^MC excited state, as demonstrated previously for [Fe(bpy)_3_]^2+^. For both complexes, relaxation from the MLCT excited state to the ^5^MC excited state occurs through a short lived ^3^MC transient with very similar rate constants.^12^ The stepwise change in the Fe spin moment during spin crossover, rather than a direct transition from the MLCT to the ^5^MC state, indicates that the sequential transitions involving single electronic transitions coupled by a spin-orbit operator have larger coupling matrix elements than the coupling for the direct transition involving the simultaneous transition of two distinct electrons on two centers. These findings are consistent with the computational studies of [Fe(bpy)_3_]^2+^ by Sousa *et al.*,^29,30^ where the sequential mechanism is predicted to result in significantly faster spin crossover than the direct mechanism. No such theoretical study has been performed on [Fe(bpy)_2_(CN)_2_]. Additional ultrafast XANES^15^ measurements on [Fe(bpy)_3_]^2+^ following the work of Zhang *et al.* have allowed the refinement of the spin crossover mechanism, demonstrating the limitations of a kinetic description of the spin crossover dynamics, but still supporting the sequential mechanism for photo-induced spin crossover. The necessity of a dynamical model for photo-induced spin crossover in [Fe(bpy)_2_(CN)_2_] seems plausible, but these ultrafast XES and UV-visible measurements lack sensitivity to these details. Most likely further ultrafast XANES, XES, and XDS measurements would enable us to resolve these issues.

This study provides an extension of our investigations of MLCT excited state relaxation in [Fe(bpy)_N_(CN)_6–2N_]^2N-4^, where N = 1–3, a series of Fe(II) complexes.^12,34^ The motivation for these studies is the systematic identification of how symmetry, ligand field strength, and covalency dictate the dynamics and mechanisms of internal conversion and intersystem crossing in 3d transition metal complexes. As expected, this series of complexes has proven to lead to a large variety of relaxation pathways. At present, we have a firm understanding of the electronic excited states involved in MLCT excited state relaxation and have demonstrated that simple ligand substitution can modify MLCT lifetimes by more than two-orders of magnitude. Further progress requires determining which vibrational motions promote the initial MLCT → ^3^MC transition. The detailed understanding of electronic excited state relaxation in 3*d* transition metal based systems remains an important pathway to the rational design of Fe(II) photocatalytic complexes with the significantly longer MLCT lifetimes needed for this application.

## SUPPLEMENTARY MATERIAL

See supplementary material for the experimental conditions and data analysis.
